# Metacognitive Therapy for Posttraumatic Stress Disorder in Youth: A Feasibility Study

**DOI:** 10.3389/fpsyg.2019.00264

**Published:** 2019-02-19

**Authors:** Michael Simons, Anna-Lena Kursawe

**Affiliations:** Department of Child and Adolescent Psychiatry, Psychosomatics and Psychotherapy RWTH Aachen University, Aachen, Germany

**Keywords:** adolescents, children, feasibility study, metacognitive therapy, posttraumatic stress disorder

## Abstract

Metacognitive therapy (MCT) is an effective treatment for posttraumatic stress disorders (PTSD) in adults. However, there is no evidence for the feasibility, acceptability, and efficacy of MCT for PTSD in youth so far. This study is the first to utilize MCT for children and adolescents with PTSD. Twenty-one children and adolescents (aged 8–19 years) who were consecutively referred to the outpatient trauma clinic were treated with MCT. In all patients, treatment was well accepted and regularly attended. At post-treatment, MCT was associated with significant and large reductions in posttraumatic stress symptoms. Depending on the outcome measure, 95 or 85% of the patients were classified as recovered after treatment. Eighteen patients were included in the calculation of the overall outcome. Effect sizes on primary PTSD measures were large (Cohen’s *d* = 3.42 and *d* = 1.92) and more than comparable to well-established treatments. Only six patients were available at follow-up, but their improvements were found to be stable. Despite the limitations of this uncontrolled study, the results suggest that MCT may be a feasible and promising treatment for traumatized children and adolescents and they justify a controlled trial evaluating the efficacy of MCT versus an already well-established intervention.

## Introduction

Many children and adolescents experience traumatic events with the potential to impact their lives substantially. About 16% of youths subjected to a traumatic event develop posttraumatic stress disorder (PTSD) (Alisic et al., 2014). A recent meta-analysis shows that PTSD prevalence reduces by approximately 50% over the first 6 months after a traumatic event, while there is little evidence of further change in prevalence or symptom severity after 6 months. This suggests that it is increasingly unlikely for a child to lose a PTSD diagnosis without intervention beyond this point (Hiller et al., 2016). In the Diagnostic and Statistical Manual of Mental Disorders (4th ed., DSM-IV-TR; American Psychiatric Association [APA], 2000), PTSD is conceptualized as symptom clusters of intrusive re-experiencing, pervasive avoidance, and hyperarousal in the aftermath of at least one traumatic event. In DSM-5 (American Psychiatric Association [APA], 2013), a fourth symptom cluster has incorporated negative alterations in cognitions and mood.

A recent meta-analysis (Morina et al., 2016) and a recent review (Dorsey et al., 2017) showed that trauma-focused treatment, especially trauma-focused cognitive-behavioral therapy (Tf-CBT), was most effective when treating PTSD in children and adolescents. In comparison to a waitlist condition, Tf-CBT was superior (Hedges’s *g* = 1.44). Tf-CBT usually includes approximately 10–12 parallel, mostly separate child and parent sessions. A recent German multicenter study found a modest effect size (Cohen’s *d* = 0.50) of Tf-CBT (12 weekly 90-min parallel or conjoint sessions with patients and caregivers) against a waitlist condition and a large within-group effect size of Tf-CBT (*d* = 1.51) (Goldbeck et al., 2016). The core element of this treatment is imaginal exposure, i.e., helping the patient recall traumatic events in detail and re-experiencing it. This kind of exposure is aimed at counteracting the patient’s avoidance of distressing thoughts or trauma reminders to reduce anxiety by habituation. Further, as conceptualized by cognitive theorists (e.g., Ehlers and Clark, 2000), this strategy aims to integrate fragmented trauma memories into autobiographical memory. Surprisingly, explicit exposure does not moderate outcomes for posttraumatic stress or depressive symptoms (Dorsey et al., 2017).

Moreover, and in line with the new conceptualization of PTSD in DSM-5, cognitive changes are important targets in CBT. Thus, besides reducing cognitive and behavioral avoidance, Tf-CBT aims to correct dysfunctional cognitions about the self, the world, and the future that patients have developed after the traumatic event. In a recent meta-analysis, Diehle et al. (2014) show that Tf-CBT leads to a larger reduction in posttraumatic stress symptoms and trauma-related cognitions than non-active and active control conditions. Exposure therapy seems to be more efficacious than cognitive interventions without exposure, while cognitive restructuring has small advantages over treatments without cognitive restructuring.

Although current practice parameters recommend trauma-focused treatments, a recent study by Wells et al. (2015) challenges this recommendation. The authors found a new, non-trauma-focused treatment approach, namely metacognitive therapy (MCT), to be superior to established trauma-focused treatments (i.e., prolonged exposure, PE) when treating PTSD in adults (Hedges’s *g* = 4.52 for MCT vs. *g* = 1.53 for PE). According to the originator of MCT (Wells, 2009), cognitions (e.g., memory structure), and general beliefs are less crucial for the development of PTSD than cognitive processes such as thought suppression, rumination, worrying, and gap filling (i.e., trying to fill in gaps in the memory). Together with dysfunctional attentional and avoidant coping strategies, these thinking processes make up the so-called cognitive attentional syndrome (CAS). These maladaptive processes are motivated by metacognitive beliefs, i.e., beliefs about thinking. Positive metacognitive beliefs motivate these processes, e.g., “I have to get rid of these thoughts in order to stop me from going mad,” “Worrying keeps me safe,” “I have to think about the event in order to find out what I could have done to prevent it from happening,” or “In order to cope with the event I have to remember it in every detail.” Negative metacognitive beliefs refer to the uncontrollability and dangerousness of thinking, e.g., “I cannot stop worrying” or “I will go crazy if I cannot stop thinking about the event.” Consistent with this model, Fergus and Bardeen (2017) found evidence that *metacognitive* beliefs, not *cognitive* beliefs, maintain posttraumatic stress. Further, Bennett and Wells (2010) found evidence that metacognitive beliefs about the trauma memory (e.g., the belief that gaps in the memory mean I am not normal), but not memory disorganization within the trauma narrative, positively predicted significant variance in posttraumatic stress symptoms. Further, Bardeen and Fergus (2018) found that deficits in executive control strengthened the positive association between metacognitive beliefs and posttraumatic stress symptoms. Wells (2009) emphasizes that the processes of the CAS are maladaptive in that they increase and maintain threat perceptions and block emotional processing. Thus, MCT aims to reduce the CAS and to modify the metacognitive beliefs which maintain it.

There is some evidence that MCT is applicable and might be efficacious in youths with obsessive-compulsive disorder (Simons et al., 2006) and with generalized anxiety disorder (Esbjørn et al., 2018). The present study describes the first attempt to treat traumatized minors with MCT with the aim to test the applicability and the feasibility of MCT for this age group. The main research question was to determine whether the established MCT manual which was developed for adults with the diagnosis of PTSD could be applied to traumatized youths. Therefore, we aimed to determine the number of patients who completed therapy regularly, the number of sessions required, and the magnitude of symptom reduction as an indication of the possible efficacy of this treatment.

## Materials and Methods

### Participants

Twenty-one children and adolescents (aged 8–19 years), who presented consecutively in the outpatient trauma clinic and who met criteria for PTSD according to DSM-IV/ICD-10 (World Health Organization [WHO], 1992; American Psychiatric Association [APA], 2000), were included in this study (see Table 1). Traumatic events comprised violent or sexual assaults, robbery, suicide of a relative, house fire, or car accident. The interval between trauma and commencement of treatment ranged from one to more than 48 months. Two of the three cases with greater than a 48-month-interval were female adolescents with long-term experiences of repeated sexual abuse and assault. One girl suffered from the aftermath of a sexual abuse event 11 years prior. In every case, the first diagnostic appointment followed no later than one week after the families’ request. Therapy began shortly after the completion of the initial assessment. The wide interval between the traumatic incident and the beginning of treatment was due to the families’ decision of when to access the outpatient clinic.

**Table 1:** Age, sex, type of trauma, duration (i.e., time between the traumatic event and the first appointment in the outpatient trauma clinic), and comorbidity.

#	Age	Sex	Type of trauma	Duration (months)	Comorbidity
1	8	F	House fire	6	ADHD
2	13	F	Sexual abuse	>18	Obesity
3	15	F	Sexual abuse	>48	Dissociative stupor
4	18	F	Suicide of brother	3	
5	16	F	Suicide attempt of boyfriend	2	
6	15	M	Sexual abuse	>36	
7	15	M	Sexual abuse	>36	
8	16	F	Rape	16	Depression
9	13	F	Domestic violence	2	
10	16	F	Sexual abuse	11 years earlier	
11	10	F	Sexual abuse	8	ADHD
12	13	F	Peer violence	1	
13	14	M	Death of family member	2	
14	13	F	Car accident	3	GAD
15	15	F	Rape	3	
16	13	F	Sexual abuse	4	Depression
17	19	F	Sexual abuse	7	
18	17	F	Sexual abuse over 4 years	>48	
19	15	F	Sexual abuse	11	Depression
20	15	F	Sexual abuse	1	
21	16	F	Sexual abuse	>48	Depression


*F, Female; M, Male; ADHD, Attention Deficit/Hyperactivity Disorder; GAD, Generalized Anxiety Disorder*.

The diagnosis of PTSD was confirmed by means of a well-established structured interview (see below). Comorbid diagnoses/problems included attention deficit hyperactivity disorder (*n* = 2), depression (*n* = 4), obesity (*n* = 1), self-harming behavior (*n* = 1), and generalized anxiety disorder (*n* = 1). None of the patients included had comorbid problems of alcohol/drug dependency and none had previously obtained any cognitive-behavioral treatment or received pharmacotherapy. All participants provided informed consent and the study was approved by the ethics committee at the RWTH Aachen Faculty of Medicine (EK 240/18).

### Measures

*The Clinician-Administered PTSD Scale for Children and Adolescents* (CAPS-CA; German version: Steil and Füchsel, 2006) is a semi-structured clinical interview designed to assess PTSD symptoms according to *DSM-IV* and *ICD-10* and associated symptoms in children and adolescents. It consists of 36 questions based on a specific event the child identifies as most distressing. The diagnosis also incorporates a clinical judgment regarding the type of trauma and impact on functioning. The CAPS-CA was administered only before treatment to confirm the diagnosis of PTSD.

Primary outcome is feasibility, that is the proportion of patients who were offered treatment who completed and the number of sessions attended. Secondary outcome was the change in posttraumatic symptoms, self-rated by the patients. These were measured at pre- and post-treatments, as well as at a follow-up 3 to 5 months after the completion of therapy with the following measures:

*The Revised Child Impact of Events Scale* (CRIES-13) is a 13-item scale measuring posttraumatic intrusion, avoidance, and hyperarousal. Items are answered on a four-point Likert-scale (0 = *not at all*, 1 = *rarely*, 3 = *sometimes*, 5 = *often*). The total score ranges between 0 and 75 with a cut-off score at 30 suggesting a probable diagnosis of PTSD (Perrin et al., 2005).

*The Child PTSD Symptom Scale* (CPSS) consists of 17 symptom items that are answered on a four-point scale from 0 (*not at all*) to 3 (*5 or more times a week*); thus, the total score ranges between 0 and 51 with a cut-off score at 11 indicating more than mild posttraumatic stress and a score of 19.1 indicating moderate posttraumatic stress (Foa et al., 2001). Note that other studies found different cut-off scores to be the optimal cut-point for the highest specificity, e.g., 16 (Nixon et al., 2013) and 21.5 (Hukkelberg et al., 2014). Seven further items assessing impairment in functioning were not analyzed in this study. We decided to apply both measures because of their respective advantages: the CRIES is a well-established and an easy comprehensible measure with the disadvantage that it does not refer to the DSM-IV. The CPSS might be a little less comprehensible but refers explicitly to the DSM-IV which was the relevant classification system at the time the therapies were conducted.

In the first 11 patients, measures were administered only before and after treatment. Beginning with the twelfth patient, we planned a further follow-up administration. All outcome measures show good retest-reliability: CRIES rtt = 0.85 (Verlinden et al., 2014), CPSS rtt = 0.84 (Foa et al., 2001).

### Intervention

The treatment was conducted by a clinical psychologist with extensive training in MCT (the first author in this study) and followed the manual developed and published by Wells and Sembi (2004) and Wells (2009) with only slight adaptions for the younger patients. It comprised of up to 14 sessions, each of about 40 to 50 min duration. An involvement of the parents in the treatment was not planned and only done if deemed necessary. Treatment was terminated when the patient and the therapist agreed that all the treatment goals (i.e., significant reduction of posttraumatic stress symptoms and resulting functional impairment) were achieved. The treatment started with a joint case formulation and becoming acquainted with the metacognitive model. Patients were introduced to the idea that the processing of a traumatic event is largely automatic, like the healing of a wound. However, the healing of a wound can be painful (itchy) and distressing and some people tend to scratch the wound which hampers the healing process. Likewise, some traumatized people utilize what they see as healing strategies like thought suppression, worrying, rumination, gap filling, threat monitoring, avoidance, and other behavioral strategies. Thus, treatment aimed to reduce and undo these unhelpful coping strategies. Thought suppression experiments (like: “Please, try not to think about a pink rabbit sitting on my head!”) were conducted to demonstrate its paradoxical effect: When an individual tries to suppress specific thoughts, the frequency of these thoughts increases and becomes more accessible than before (“rebound effect,” Wegner et al., 1987). Patients were introduced to new strategies in dealing with intrusive thoughts/memories. First, they learned to leave the thoughts alone (“detached mindfulness”). This was explained using analogies like the telephone metaphor: “You cannot decide when the phone rings, but you can learn to let it ring without picking up. Further, if the caller left a message on the answering machine/mailbox, you can deal with it later. Similarly, it is not your decision when these thoughts pop into your mind, but you can learn to leave these thoughts alone and deal with them later.” Likewise, patients learned to postpone worrying and rumination to a fixed time in the early evening which should not exceed 10 min. These postponement experiments aim to weaken metacognitive beliefs about the uncontrollability of worrying and rumination. Experiments and verbal strategies were used to challenge further negative and positive metacognitive beliefs about worrying and rumination. The treatment continued with attention modification experiments to reduce threat monitoring, oftentimes combined with being in social situations. For example patients were asked to enter situations they had avoided since the traumatic event while focusing their attention on the safe aspects of the situation. Treatment was terminated after discussing relapse prevention strategies. Cognitive behavioral strategies, like imaginal reliving or challenging of thoughts and beliefs about trauma, or repeated exposure *in vivo* with the aim of habituation, were not conducted. Patients were invited to talk about the traumatic event if they so wished, but in fact no one made use of this offer.

### Data Analyses

Analyses were performed using the Statistical Package for Social Sciences (SPSS, version 23.0). Raw scores were used for all analyses and the critical alpha level was set at 0.05. Due to the different numbers of questionnaires available at post-treatment and follow-up, sample sizes differed for each measure and time-point of measurement administration. Thus, analyses were performed separately for each questionnaire and time-point. The main analyses were comprised of paired-sample *t*-tests on data of the CRIES and CPSS for pre-post-treatment comparisons, while the non-parametric Wilcoxon Signed-Ranks test was used on comparisons of post-treatment data with follow-up data on the CRIES and CPSS because of the small sample sizes. Corrected effect sizes of Cohen’s *d* for significant effects of the treatment effects analyses (Hedges and Olkin, 1985; Cohen, 1988; Cumming and Finch, 2005) and reliable change indices (RCI) were documented for each participant to discover clinically meaningful changes in the individual score beyond measurement error^1^.

To calculate the effect size, we used the formula of Cohen’s *d* = *M*_1_–*M*_2_/*SD_Pooled_*. To calculate the RCI, we subtracted the post-treatment score from the pre-treatment score and divided the result by the standard error of the difference between the two scores, which was calculated using standard deviations of the current sample and reliability coefficients of the test instrument [RCI = (posttest – pretest)/SEM)] An RCI value greater than 1.96 is considered a clinically significant improvement ( RCI > 1.96: “improved,” -1.96 < RCI < 1.96: “unchanged;” RCI < -1.96: “impaired;” Jacobson and Truax, 1991). As a further criterion of clinically significant change, we investigated if the scores of the CRIES and the CPSS fell below the cut-off scores. Using the RCI and the cut-off points, each patient could be classified as recovered (passed both criteria), improved (passed only the RCI criterion in the positive direction), unchanged (did not pass the RCI criterion), or impaired (passed the RCI criterion in the negative direction).

## Results

All patients entering the study completed treatment; one patient who did not benefit from treatment was referred to inpatient therapy after completion. Treatment was rather short with an average of 7 sessions (range 3–14). Even the youngest patient, an 8 year old girl with chronic PTSD after house fire, completed therapy successfully after only five sessions. Treatment was shorter when specific processes, especially gap filling, or positive metacognitive beliefs could not be identified and thus were not in need of change. Although we did not collect data on parent’s involvement, we can state that they were involved very rarely. As can be seen in Table 2, we were able to obtain pre to post treatment data on at least one outcome measure for all of the 21 patients.

**Table 2:** Descriptive statistics on the main outcome measures at pre-treatment, post-treatment and follow-up and reliable change indexes.

#	Number of sessions	CRIES_Pre_	CRIES_Post_	RCI_CRIES_	CRIES_FU_	CPSS_Pre_	CPSS_Post_	RCI_CPSS_	CPSS_FU_
1	5	31	12^∗^	–	–	–	–	–	–
2	6	40	3^∗^	7,02	–	12	2^∗^	1,76	–
3	10	–	–	–	–	22	0^∗^	3,86	–
4	10	–	–	–	–	25	7^∗^	3,16	–
5	3	45	7^∗^	7,21	–	31	1^∗^	5,27	–
6	6	49	0^∗^	9,30	–	18	0^∗^	3,16	–
7	6	48	0^∗^	9,11	–	19	0^∗^	3,34	–
8	14	62	53	1,71	–	43	48	-0,88	–
9	8	43	12^∗^	5,88	–	34	3^∗^	5,44	–
10	4	36	2^∗^	6,45	–	27	3^∗^	4,21	–
11	3	35	2^∗^	6,26	–	21	3^∗^	3,16	–
12	4	37	3^∗^	6,45	2	11	1^∗^	1,76	1
13	4	57	16^∗^	7,78	–	36	8^∗^	4,92	–
14	7	36	7^∗^	5,50	12	17	2^∗^	2,63	11
15	10	38	13^∗^	4,74	–	22	5^∗^	2,98	–
16	14	51	6^∗^	8,54	15	25	8^∗^	2,98	6
17	10	61	8^∗^	10,06	–	33	2^∗^	5,44	–
18	11	55	3^∗^	9,87	3	40	1^∗^	6,85	1
19	7	61	23^∗^	7,21	7	39	9^∗^	5,27	6
20	8	41	3^∗^	7,21	0	18	5^∗^	2,28	0
21	7	57	2^∗^	10,44	2	37	2^∗^	6,14	1


*CRIES, Children’s Revised Impact of Events Scale; CPSS, Child PTSD Symptom Scale; RCI, Reliable Change Index; Pre, Pre-treatment; Post, Post-treatment; FU, follow-up. ^∗^Scores below the cut-off point*.

Because of incomplete data sets due to administrative error (i.e., measures not given), three patients had to be excluded from pre-post calculations on CRIES and CPSS. Thus, analysis on CRIES and CPSS contained 18 patients (14 females, mean age 14.67, range 10–19 years) Further, because we started to collect follow-up data relatively late in the course of the study, data exists of only 6 patients (all female, mean age 14.33, range 13–17 years) for the CRIES and CPSS.

Table 3 presents the means and standard deviations of each outcome measure at pre-treatment and post-treatment, and also the effect size (Cohen’s *d*) from pre- to post-treatment.

**Table 3:** Means and standard deviations for outcome measures at pre-treatment, post-treatment, and follow-up, Cohen’s *d* for pre- to post-treatment.

Measure	Pre		Post		Cohen’s *d pre post*	FU	
	*M*	*SD*	*M*	*SD*		*M*	*SD*
CRIES	47.33	9.62	9.06	12.55		6.50	5.96
N_Pre,Post_= 18,					3.42		
N_FU_= 6							
CPSS	26.83	10.07	5.72	10.90		4.17	4.26
N_Pre,Post_= 18,					1.92		
N_FU_= 6							


*CRIES, Children’s Revised Impact of Events Scale; CPSS, Child PTSD Symptom Scale; M, Mean; SD, Standard Deviation; Pre, Pre-treatment; Post, Post-treatment; FU, Follow-up*.

### Pre-post Treatment Effects

*t*-Tests indicate that patients’ symptoms improved significantly from pre- to post-treatment (CRIES: *t*(17) = 14.32, *p* < 0.001, *d* = -3.42; CPSS: *t*(17) = 8.23, *p* < 0.001, *d* = -1.92).

### Clinical Significance

At post-treatment, all but one of the 21 patients scored below the cut-off and no longer met the diagnostic criteria for PTSD. In regard to the individual RCI (see Table 2), clinically significant improvement was found in 18 out of 19 patients (CRIES), and in 17 out of 20 patients (CPSS). Thus, depending on the outcome measure, 95% (CRIES) and 85% (CPSS) met criteria for recovery, whereas one patient (subject 8) was found to be unchanged (CRIES) and impaired (CPSS).

### Follow-Up Treatment Effects

For both outcome measures of the CRIES and CPSS, Wilcoxon Signed-Ranks tests did not reveal significant differences (CRIES: *Mdn_Post_* = 4.5, *Mdn_FU_* = 5.0, *Z* = -0.14, *p* = 0.89; *Mdn_Post_* = 3.5, *Mdn_FU_* = 3.5, CPSS: *Z* = -0.37, *p* = 0.72), indicating that improvement in PTSD symptoms was maintained from post-treatment to the follow-up at least in the 6 cases measured over this time frame.

## Discussion

This is the first study that aimed to test the feasibility, acceptability, and effects associated with MCT in the treatment of PTSD in youths. The results show that treatment was feasible as indexed by all patients completing the course. In addition, the duration of treatment was within the range recommended for adults with MCT. Treatment was associated with clinically significant effects in posttraumatic stress symptoms in almost all participants. Effect sizes were large (Cohen’s *d* = 3.42 in CRIES, *d* = 1.92 in CPSS) and seemingly higher than effect sizes reported in a study of Tf-CBT (Goldbeck et al., 2016). Depending on the outcome measure, 85 or 95% of patients were found to have recovered. In all 6 patients available for the follow-up, the improvement was maintained.

It appears that MCT treatment can be delivered to traumatized youths in a small number of sessions (mean = 7, range 3–14) of 40–50 min duration each and it is associated with large symptom improvements. This compares favorably with Tf-CBT which is usually conducted over 10–12 sessions each lasting 90 min. Thus, MCT might be more time effective than Tf-CBT, but this remains to be directly tested. The results demonstrate the feasibility and possible efficacy of MCT and add to the recent literature evaluating MCT for PTSD in adults (Wells and Colbear, 2012; Wells et al., 2015). Further, if replicated, the results may have some important implications regarding the mediators of psychotherapy. First, an efficacious psychotherapy for PTSD may not have to be trauma-focused (i.e., imaginal reliving). To reduce posttraumatic intrusions, it may be sufficient to stop the efforts to suppress these thoughts as is practiced in detached mindfulness, and to reduce extended thinking processes.

The limitations of this study are obvious; it is an uncontrolled study with a single therapist. Furthermore, at follow-up, only six patients were available. Measures of metacognition, anxiety, and depression were not included and neither were parent reported outcomes. Because of the small sample size, moderators of treatment efficacy like comorbidity or type of traumatic event were not examined. Further, stable pre-treatment baselines were not established. Thus, improvement could also be attributed to spontaneous remission. However, twelve patients (57%) suffered more than 6 months from PTSD which makes spontaneous remission rather improbable (Hiller et al., 2016). However, we cannot partial the effects of treatment from other possible influences on symptom change.

Despite these major limitations, the results show that a course of MCT treatment could be implemented with children and adolescents suffering from PTSD over a course consistent with adult treatment. The results signal the need for a better controlled study (i.e., randomization, blind assessors, different therapists, etc.,), testing MCT against a well-established treatment of PTSD, like Tf-CBT or Eye Movement Desensitization and Reprocessing (EMDR). An investigation of the importance of changes in cognitions and metacognitions in the efficacy of treatment would also be of further interest.

## Author Contributions

MS initiated the project and conducted assessment and therapy. A-LK analyzed the data and wrote the passages of data analyses and results.

## Conflict of Interest Statement

The authors declare that the research was conducted in the absence of any commercial or financial relationships that could be construed as a potential conflict of interest.

## Footnotes

1 The retest-reliability for each test to compute the RCI scores have been previously reported (Foa et al., 2001; Verlinden et al., 2014).
